# Hypertonic sodium lactate improves microcirculation, modulates inflammation, and preserves vascular reactivity in a rat model of sepsis: *in vivo *and* ex vivo* evidence

**DOI:** 10.1186/s40635-026-00975-5

**Published:** 2026-09-20

**Authors:** Eva Correia, Thomas Maudhuizon, Antoine Hérault, Soumeya Bekri, Eric Fontaine, Jérémy Bellien, Paul Mulder, Fabienne Tamion, Emmanuel Besnier

**Affiliations:** 1 Univ Rouen Normandie, Inserm, EnVI UMR 1096, Rouen, F-76000 France; 2 Departement of Medical Intensive Care Unit, CHU Rouen, Rouen, F-76000 France; 3 Department of Metabolic Biochemistry, Referral Center for Lysosomal Diseases, UNIROUEN, AIMS, CHU Rouen, Normandie University, Rouen, G2M SysMedLab France; 4 Department of Endocrinology, Diabetology and Nutrition, CHU Grenoble Alpes, Inserm U1055, LBFA, Université Grenoble Alpes, Grenoble, France; 5 Department of Pharmacology, CHU Rouen, Rouen, F-76000 France; 6 Department of Anesthesia and Critical Care, CHU Rouen, Rouen, F-76000 France

**Keywords:** Sepsis, Hypertonic sodium lactate, Microcirculation, Cardiovascular dysfunction

## Abstract

**Background:**

Hypertonic sodium lactate (HSL) has demonstrated beneficial effects in experimental sepsis, improving hemodynamics, modulating inflammation, and supporting cellular metabolism. Nevertheless, prior studies have not specifically addressed microcirculation, despite its critical impairment during sepsis and the absence of targeted therapies. We evaluated the therapeutic effects of HSL in a in vivo resuscitated rat model (Male wistars) of cecal ligation and puncture (CLP), with a particular focus on microcirculatory function, followed by ex vivo investigation of sodium lactate on vascular reactivity.

**Results:**

10 h after CLP rats were infused with 0.9% NaCl or 11.2% HSL for 18 h before exploration. No difference was observed on arterial pressure, echocardiography and pressure-volume loops. HSL improved mesenteric perfusion (1126 [923–1300] vs. 816.5 [692–919] Perfusion Unit, *p* = 0.012) and TNF-α plasma levels (26.2 [22.8–37.1] vs. 42.6 [32.6–47.3] pg/mL, *p* = 0.001). Ex vivo, mesenteric artery reactivity experiments from healthy rats was tested with or without LPS-stimulation and glucose deprivation. Lactate dose-dependently restored LPS-impaired vasoconstriction, showing an independent effect on small arteries. In glucose deprivation conditions, HSL was able to restore vascular reactivity in normal or LPS-stimulated arteries.

**Conclusions:**

HSL specifically improved microcirculation and reduced systemic inflammation in septic rats. Moreover, lactate may act as an alternative to glucose in deprivation conditions. These benefits support its translational potential as a resuscitation fluid in sepsis.

**Supplementary Information:**

The online version contains supplementary material available at https://doi.org/10.1186/s40635-026-00975-5.

## Introduction

Sepsis is a significant global health concern, accounting for at least 30 million cases and 6 million deaths annually worldwide [1]. Circulation is particularly threatened, exposing patients to hypovolemia and vasodilation resulting from various and complex mechanisms [2]. Therefore, fluid infusion remains the first-line treatment to maintain blood pressure, cardiac preload and end-organ perfusion [3]. Nevertheless, sepsis induces a microvascular dysfunction, with enhanced vascular leakage that promotes water accumulation in organs, resulting in a positive fluid balance known to be associated with mortality [4–7]. Fluids resuscitation, such as unbalanced sodium chloride (NaCl 0.9%) are widely used in clinical settings, but their use is still debated because of potential side-effects [8–10] and questions remain regarding the best fluid and the appropriate strategy for its administration [11, 12]. Lactate-containing fluids may offer numerous advantages, including reduced chloride load thanks to the incorporation of lactate as an anion. Moreover, a potential energy supply from lactate and its metabolites may occur when fluids contain high lactate levels. Experimental deprivation of lactate metabolism using dichloroacetate has been shown to impair cardiac function in murine models of endotoxemic shock [13, 14]. Hypertonic sodium lactate (HSL) has already demonstrated beneficial effects in different models [15], such as experimental models of brain injury [16–18], cardiac arrest [19, 20], myocardial infarction [21], endotoxemia and even sepsis. In paediatric dengue shock syndrome, HSL infusion improved hemodynamics with limited fluid accumulation in a randomized single-blinded clinical trial [22]. In a porcine model of endotoxemia, HSL enhanced hemodynamic parameters, renal function, and microvascular flow, and has been associated with reducing systemic inflammation [23]. In our laboratory, we previously demonstrated improvements in cardiac function, systemic and mesenteric circulation, inflammation, and renal outcomes following HSL administration in a rat model of sepsis induced by cecal ligation and puncture (CLP) [24]. Nevertheless, results were inconclusive regarding microcirculation, despite its tight link with macro-circulation. This is particularly important because, during sepsis, these two systems may evolve independently. In fact, this phenomenon known as microvascular incoherence, describes a state in which macro-circulation can be restored while microcirculation remains impaired, thereby compromising organ perfusion. This unresolved discrepancy directly supports our hypothesis that lactate may act as a metabolic fuel for organs, including the microvasculature, and could therefore specifically target this dissociation. In the present study, we sought to address this gap by focusing on microcirculation and the energetic role of lactate using two separated approaches: *in vivo* and ex vivo.

## Materials and methods

### Animal procedures

We conducted a prospective, randomized, controlled experimental study approved by the Ethics Committee for Animal Research (CENOMEXA no. 54, approval number #32551-2021072614374803, 16 February 2022, and #45309-2023102511082103, 29 April 2024). All procedures were performed in accordance with the French Ethics Committee, the guidelines of the European Parliament directive 2010/63/EU, and the Council for the Protection of Animals Used for Scientific Purposes. The elaboration of this manuscript adheres to the ARRIVE guidelines 2.0 [25]. Procedures were previously published and are briefly described in the following sections [24].

Number of animals per experiments was based on previous experiments, with *n* = 9 per group in most of procedures, except for invasive cardiac hemodynamic (*n* = 7) and arteriography (*n* = 5). Because of the high variabilities between operators and the great modification in the protocol (antibiotics, post-CLP infusion etc.), previous data from our unit would not have been transposable to this protocol, and therefore calculating an a priori sample size would have been unreliable. Animals were randomly assigned to CLP-NaCl or CLP-HSL, except for a set of experiments dedicated to determining the impact of CLP on circulation, where a Sham group and a CLP group without fluids were also randomly constituted, to characterize the model. In other experiments, these Sham and CLP groups were not included to minimize the use of animals, because we previously published data on the beneficial effect of HSL, and according to ethical considerations. To reduce animal use and maximize data extraction, some experiments were structured to allow assessment of multiple endpoints within the same animal.

A first set, including four groups (Sham, CLP, CLP-NaCl and CLP-HSL) was dedicated to characterizing the impact of CLP and fluids on circulation by using telemetry monitoring of heart rate and blood pressure. This first set allowed to determine the optimal time-point for fluid infusion and antibiotics therapy, as we considered that the initiation of blood pressure drop mimics the onset of sepsis in rats.

A second set was dedicated to characterizing cardiac effects using echocardiography and invasive left ventricle catheterization (CLP-NaCl and CLP-HSL groups). Finally, a third set evaluated *in vivo* mesenteric microcirculation and biochemical analyses (CLP-NaCl and CLP-HSL groups).

### Telemetry assessment

Telemetry was performed on male Wistar rats aged 12-weeks-old (Janvier Labs, France), after at least one week of acclimatization. Rats were anesthetized by inhaled isoflurane (2.5%) (Isovet^®^, B. Braun, France) and intraperitoneal injection of xylazine (5 mg/kg) (Sedaxylan^®^ 20 mg/mL, Dechra, France) one day before being randomly submitted to Sham or CLP surgery as described below. The monitoring of blood pressure and heart rate was assessed via telemetry (DSI^®^ Data Sciences International System, USA). The telemetric implant catheter was placed in the left femoral artery, and the sensor was positioned subcutaneously at the dorsal level. The skin incision was then closed using 4 − 0 Vicryl sutures (Ethicon, USA). Signals were recorded in conscious animals from 24 h before sepsis induction until sacrifice. Data were acquired every 30 min and analysed using Dataquest A.R.T.™ Gold Acquisition (Version 4.36).

#### Animal model of sepsis

Sepsis was induced by CLP 24 h after at least one week of acclimatization, or after 24 h of telemetry procedure. Rats were anesthetized by inhaled isoflurane (2.5%) (Isovet^®^, B. Braun, France) and intraperitoneal injection of xylazine (5 mg/kg) (Sedaxylan^®^ 20 mg/mL, Dechra, France). After a 2 cm cervical incision, a 3-F polyurethane perfusion catheter was inserted in the right jugular vein. The catheter was tunneled subcutaneously and connected to a swivel-based perfusion harness (Instech Laboratories^®^, USA). The skin incision was then closed using 4 − 0 Vicryl sutures (Ethicon, USA). The cecum was ligated (75% of its volume) and punctured twice (16-G needle) to externalize feces. Sham-operated rats were processed with cervicotomy and laparotomy, but no catheter introduction or CLP procedure, and served as controls for the first set only.

### Treatment

Ten hours after CLP surgery, based on the decrease of arterial pressure determine by telemetry (see results section), animals were randomly divided in 3 groups: CLP without fluid infusion and antibiotics (only for telemetry assessment), CLP-NaCl receiving 0.9% NaCl (154 mmol/L of chloride + 154 mmol/L of sodium) (Sodium Chloride, Aguettant, France) and CLP-HSL receiving 11.2% sodium lactate (1000 mmol/L of sodium + 1000 mmol/L of lactate) (APHP, France). Both types of fluids were administered at 2.5 mL/kg/hr for 18 h in association with the antibiotic piperacillin/tazobactam (KABI, France, 65 mg/kg subcutaneous).

To match the caloric intake provided by lactate, 8% sterile glucose (VWR BDH Chemicals, USA) was added to NaCl fluid as previously described [23, 24], without changing sodium concentrations.

All rats received subcutaneous tramadol (Tramadog^®^, TVM, France) injections at 20 mg/kg/12 hours for the entire duration of the protocol. CLP-NaCl and CLP-HSL rats did not have access to water and chow. Rats under experiment were housed individually in a cage. The experimental process and distribution of animals among the different experimental sets are presented in Annex 1.

### Transthoracic echocardiography

Echocardiography was performed under 2% isoflurane anesthesia using a Vivid 7 ultrasound system^®^ (GE Healthcare, France) with a 14 MHz M12L linear probe and Echota PC software^®^ (GE Medical Systems, France). A two-dimensional parasternal long‐axis view of the left ventricle at the papillary muscle level was used to obtain M‐mode tracings. Left ventricular (LV) end‐diastolic (EDD) and end‐systolic (ESD) diameters, as well as end‐diastolic anterior wall thickness, were measured over at least 3 consecutive cardiac cycles. LV fractional shortening (FS) was calculated as FS (%) = ((LVEDD − LVESD)/LVEDD) × 100. LV outflow diameter was measured. A five cavities apical view was then performed to obtain LV outflow velocity using pulsed‐wave Doppler, and cardiac output (CO) was calculated as CO = aortic VTI × [π × (LV outflow diameter/2)²] × heart rate, where VTI is the velocity–time integral.

#### Left ventricular hemodynamic invasive assessment

LV hemodynamics was assessed using LV pressure–volume curves. Rats were anesthetized by intraperitoneal injection of ketamine (75 mg/kg) (Anesketin^®^ 100 mg/mL, Dechra, France) and xylazine (5 mg/kg) (Sedaxylan^®^ 20 mg/mL, Dechra, France). A 2 F miniaturized combined conductance catheter–micromanometer (model SPR-838, Millar, USA), connected to a pressure-conductance unit (MPCU-200, Millar, USA), was advanced through the carotid artery into the left ventricle. Pressure–volume loops were recorded at baseline and during a gently occlusion of the abdominal aorta with a cotton swab, allowing the calculation of LV end-systolic and end-diastolic pressures, LV dP/dtmin and max, LV relaxation constant Tau (Weiss method) and LV end-systolic and end-diastolic pressure–volume relations as indicators of load-independent LV contractile function and passive compliance respectively.

### Mesenteric perfusion

Mesenteric perfusion was studied by laser speckle contrast imaging (MoorLFPI-2 Full Field Laser Perfusion Imager – Moor Instruments, UK) on the gut. Rats were anesthetized by intraperitoneal injection of ketamine (75 mg/kg) (Anesketin^®^ 100 mg/mL, Dechra, France) and xylazine (5 mg/kg) (Sedaxylan^®^ 20 mg/mL, Dechra, France). The gut was carefully exposed after reopening of laparotomy, at 20 cm from the laser source, using a resolution of 1500 frames/256 colors. Image analysis was performed in four regions of interest distant from the jejunal site, and mean values are expressed as perfusion units (PU).

### Biochemistry and biological parameters

At sacrifice, blood was sampled from the abdominal aorta allowing the quantification of the metabolic and biochemical parameters and inflammation measurement.

### Biochemical measurements

A 150 µL of arterial blood sample was collected and analysed using the epoc^®^ Blood Analysis System (Siemens Healthineers^®^, Germany), which includes the epoc^®^ Reader (Reference: 11413815, portable card reader) and the epoc^®^ Host NXS (Reference: 11413497, handheld host for data processing), along with epoc^®^ Test Cards (Reference: 10736515, single-use test cards). The following parameters were measured: pH, sodium (Na⁺), potassium (K⁺), chloride (Cl⁻), hematocrit, paCO₂, paO₂, and total CO₂ (TCO₂).

### Metabolic measurement

Blood samples were immediately placed on ice. Prior to the measurement of plasma concentrations of lactate, pyruvate, 3-hydroxybutyrate, and acetoacetate, blood samples underwent deproteinization. An equal volume of perchloric acid was added (1:1 ratio), followed by three centrifugation steps at 3500 g for 15 min each at 4 °C. The resulting supernatants were then frozen at -20 °C for storage. Lactate, pyruvate and ketone bodies were measured using c111 instrument (Roche Diagnostics, France).

#### Cytokines levels

Blood samples collected from the aorta were transferred into EDTA tubes and centrifuged at 1000 × g for 20 min. Plasma levels of interleukin-10 (IL-10), interleukin-1β (IL-1β), interleukin-6 (IL-6), and tumor necrosis factor-alpha (TNF-α) were quantified using commercial sandwich ELISA kits (Quantikine™ ELISA Immunoassays, R&D Systems^®^, Minneapolis, MN, USA), following the manufacturer’s instructions. The following kits were used: IL-10: Rat IL-10 Immunoassay (Catalog numbers R1000, SR1000, PR1000); IL-1β: Rat IL-1β/IL-1F2 Immunoassay (Catalog numbers RLB00-1, SRLB00, PRLB00); IL-6: Rat IL-6 Immunoassay (Catalog numbers R6000B, SR6000B, PR6000B); TNF-α: Rat TNF-α Immunoassay (Catalog numbers RTA00-1, SRTA00, PRTA00). Plasma samples were used undiluted for all assays.

### Arteriography

This experiment was performed on isolated mesenteric arteries from non-septic rats, and inflammation was mimicked using lipopolysaccharide (LPS). Phenylephrine-mediated constriction was evaluated on 2–3 mm segments of second-order mesenteric resistance arteries isolated from healthy Wistar rats. The mesentery was removed and placed in cold oxygenated Krebs buffer with or without glucose to evaluate the metabolic effects of lactate. The presence or absence of glucose was designed to explore the ability of lactate to serve as an alternative energy substrate for microvascular function under inflammatory conditions. Krebs solution contains: NaCl 120 mM, KCl 5 mM, CaCl₂ 2.6 mM, MgSO₄ 2.4 mM, KH₂PO₄ 2.4 mM, NaHCO₃ 24 mM, and glucose 5.9 mM (when added), all purchased from VWR BDH Chemicals (USA). Mesenteric resistance artery was isolated and mounted on an arteriograph (DMT 114P, Denmark). Vessels were constricted using 10^− 6^, 10^− 5^ and 10^− 4^ M phenylephrine (Acros Organics Janssen Pharmaceuticalaan, Belgium). An initial phenylephrine-induced constriction was performed to assess arterial vasoreactivity (control phase). Arteries showing < 30% constriction from baseline were excluded from experimental phase. Outer diameters were analysed by Myo VIEW 4 (version 4.3.1.0, 2015, DMT, Denmark). Constriction was evaluated in presence or absence of LPS at 1 µg/mL for 1 h (LPS Escherichia Coli O127:B8, Sigma life Science, USA), with or without various concentrations of sodium lactate (1, 5 or 10 mM for 15 min) (Sigma-Aldrich, USA). The experimental protocol of arteriography is presented in Annex1.

### Statistical analysis

Data are presented as median and interquartile ranges. Comparisons between groups were performed using a non-parametric test (Kruskal-Wallis or Mann-Whitney tests) or a parametric test (ANOVA, repeated-measures ANOVA, or Student’s t-test) according to distribution normality, and when significant results were observed, *post hoc* analyses were performed using either Dunn’s or Tukey’s multiple comparisons tests. All analyses were performed using Prism v8.0 (GraphPad, USA). A p-value < 0.05 was considered statistically significant.

## Results

### Determination of treatment timing

To determine the optimal timing for treatment initiation in our model, the evolution of systolic and diastolic arterial pressures, as well as heart rate, following CLP without fluid administration was monitored by telemetry. Compared to Sham, CLP significantly reduced diastolic and systolic pressures after 10 h (Sham 91 [85–98] vs. CLP 87 [65–95] mmHg, *p* = 0.001 and Sham 126 [118–135] vs. CLP 112 [95–124] mmHg, *p* = 0.001) (annex 2). This drop in arterial pressure was accompanied by a significant increase in heart rate (Sham 359 [334–402] vs. CLP 430 [356 − 75] bpm, *p* = 0.001). CLP resulted in a 24-hour survival rate of 75% (annex 2 A). The decrease in arterial pressure visually began 10 h after CLP. Based on these observations, infusion treatment with NaCl or HSL, along with antibiotic therapy, was initiated 10 h after CLP.

### Restoration of arterial pressure after fluid resuscitation

In these conditions, both CLP-NaCl and CLP-HSL groups presented an increase in arterial pressure versus CLP without fluids. Diastolic and systolic pressures were similarly restored by both treatments (DBP: CLP 86 [65–95] vs. CLP-NaCl 93 [86–100] mmHg, *p* = 0.001; and CLP 86 [65–95] vs. CLP-HSL 91 [85–96] mmHg, *p* = 0.001; SBP: CLP 112 [95–124] vs. CLP-NaCl 116 [108–122] mmHg, *p* = 0.001; and CLP 112 [95–124] vs. CLP-HSL 117 [109–125] mmHg, *p* = 0.001)) (annex 2). Restoration of arterial pressure was accompanied by a significant increase in heart rate compared with CLP animals CLP 430 [356–475] vs. CLP-NaCl 438 [408–476] bpm, *p* = 0.001; and CLP 430 [356–475] vs. CLP-HSL 451 [403–478] bpm, *p* = 0.001). Both treatments resulted in a 100% survival at 28 h (annex 2B).

Then, we compared the effects of NaCl and HSL perfusion 10 h following CLP on cardiac, vascular and biological parameters.

### Impact on cardiac parameters

Echocardiographic and LV invasive hemodynamic assessments revealed no significant differences between the CLP–NaCl and CLP–HSL groups (Tables 1 and 2).

**Table 1:** Trans-thoracic echocardiography in CLP-NaCl (*n* = 9), and CLP-HSL (*n* = 9) rats after the end of fluids infusion

	CLP-NaCl		CLP-HSL		*P* value		
					CLP-NaCl vs. CLP-HSL		
**AWd (mm)**	2.4	[2.35–2.9]	2.6	[2.4–3.2]	0.29		
**AWs (mm)**	3.4	[3.2–4.2]	4.0	[3.5–4.3]	0.31		
**LVEDD (mm)**	7.2	[6.3–7.6]	6.6	[6.0-7.4]	0.35		
**LVESD (mm)**	3.4	[3.0-4.1]	3.5	[3.1-4.0]	0.86		
**PWd (mm)**	2.6	[2.4–3.1]	2.8	[2.3–3.1]	0.98		
**PWs (mm)**	3.3	[3.1–3.9]	3.4	[3.3–3.8]	0.59		
**FS (%)**	48.0	[46.6–55.4]	46.3	[44.8–50.0]	0.21		
**VTI pulm (cm)**	5.5	[5.1–6.1]	5.5	[5.4–6.3]	0.78		
**CO (mL/min)**	160	[125–217]	152	[114–202]	0.50		
**SV (mL)**	0.34	[0.30–0.50]	0.39	[0.30–0.44]	0.78		

*P* values represent statistical results using Mann-Whitney test. Awd: Anterior wall thickness at diastole. Aws: Anterior wall thickness at systole. CLP: Caecal ligation and puncture. CO: Cardiac output. FS: Fractional shortening. HSL: Hypertonic sodium lactate. LVEDD: Left ventricular end diameter at diastole. LVESD: Left ventricular end diameter at systole. NaCl: Sodium chloride. PWd: Posterior wall thickness at diastole. PWs: Posterior wall thickness at systole. SV: Stroke volume. VTI: Velocity–time integral

**Table 2:** Invasive hemodynamic parameters and pressure-volume loops in CLP-NaCl (*n* = 7) and CLP-HSL (*n* = 7) rats

	CLP-NaCl		CLP-HSL		*P* value
					CLP-NaCl vs. CLP-HSL
**LVEDP (mmHg)**	5.3	[5.1-8.0]	5.0	[4.4-6.0]	0.27
**LVESP (mmHg)**	104.2	[98.0-111.3]	94.1	[84.4–113.0]	0.33
**dP/dt**_**min**_ **(mmHg/s)**	7963	[6899–9173]	7963	[6860–9111]	0.93
**dP/dt**_**max**_ **(mmHg/s)**	7533	[6607–8038]	7230	[5821–8949]	0.83
**Tau (ms)**	9.5	[8.5–10.2]	9.1	[7.4–10.2]	0.84
**LVEDVR (mmHg/RVU)**	2.9	[1.8–4.6]	2.8	[2.1–3.4]	0.73
**LVESVR (mmHg/RVU)**	18.20	[15.0-22.4]	22.3	[15.8–39.5]	0.27

*P* values represent statistical results using Mann-Whitney test. CLP: Caecal ligation and puncture. HSL: hypertonic sodium lactate. LVEDP: Left ventricular end diastolic pressure. LVESP: Left ventricular end systolic pressure. LVEDPVR: left ventricular end-diastolic pressure-volume relationship. LVESPVR: left ventricular end-systolic pressure-volume relationship. NaCl: sodium chloride. Tau: isovolumic relaxation constant

### Biologic parameters

#### Biochemistry

Results are presented in Table 3. Compared with CLP–NaCl animals, the CLP–HSL group exhibited significantly lower plasma potassium (2.8 [2.6–3.4] vs. 3.7 [3.6–4.1] mmol/L, *p* = 0.005) and chloride levels (100 [98–103] vs. 105 [102–106] mmol/L, *p* = 0.045), while sodium concentration was significantly higher (147 [145–150] vs. 144 [142–146] mmol/L, *p* = 0.048).

**Table 3:** Biological parameters in CLP-NaCl (*n* = 9) and CLP-HSL (*n* = 9) rats

	CLP-NaCl	CLP-HSL	*P* value
			CLP-NaCl vs. CLP-HSL
**Blood ionogram**			
K^+^ (mmol/L)	3.7 [3.6–4.1]	2.8 [2.6–3.4]	0.005
Na^+^ (mmol/L)	144 [142–146]	147 [145–150]	0.048
Cl^−^ (mmol/L)	105 [102–106]	100 [98–103]	0.045
**Blood gases**			
PaCO_2_ (mmHg)	56.5 [44.7–58.6]	61.3 [45.1–64.9]	0.094
PaO_2_ (mmHg)	58.6 [53.2–75.9]	59.6 [57.5–83.3]	0.44
TCO_2_ (mmHg)	29.8 [28.9–31.5]	35.9 [31.8–40.1]	0.004
pH	7.35 [7.33–7.41]	7.38 [7.36–7.46]	0.108
**Blood metabolic parameters**			
Glucose (mg/dL)	188 [179–240]	231 [171–279]	0.37
Lactate (mmol/L)	1.46 [1.15–3.05]	1.09 [0.90–1.44]	0.16
Pyruvate (µmol/L)	163 [111–217]	110 [79–145]	0.062
Lactate/pyruvate	9.8 [7.8–15.7]	9.5 [8.9–11.7]	0.86
3-Hydroxybutyrate (µmol/L)	84 [76–194]	99 [60–195}	0.59
Acetoacetate (µmol/L)	65 [52–135]	115 [97–241]	0.040
Hematocrit (%)	41.0 [39.0-44.5]	45.0 [42.0–47.0]	0.037

*P* values represent statistical results using Mann-Whitney test. CLP: Caecal ligation and puncture. Cl⁻: Chloride. HSL: hypertonic sodium lactate. K⁺: Potassium. Na⁺: Sodium. NaCl: Sodium chloride, PaCO₂: Partial pressure of carbon dioxide. PaO₂: Partial pressure of oxygen. pH: Potential of hydrogen. TCO₂: Total carbon dioxide

Regarding blood gases, partial pressure of oxygen (PaO₂) and carbon dioxide (PaCO₂) values did not differ significantly between groups, whereas total CO₂ content (TCO₂) was higher in the CLP–HSL group (35.9 [31.8–40.1] vs. 29.8 [28.9–31.5] mmHg, *p* = 0.004), without affecting arterial pH.

No significant differences were observed in blood glucose, lactate, pyruvate, or the lactate-to-pyruvate ratio. However, CLP–HSL animals showed a marked increase in acetoacetate (115 [97–241] vs. 65 [52–135] µmol/L, *p* = 0.040). Similarly, hematocrit was significantly higher in the HSL-treated group (45.0 [42.0–47.0] % vs. 41.0 [39.0–44.5] %, *p* = 0.037).

#### Cytokines levels

**Fig. 1:**
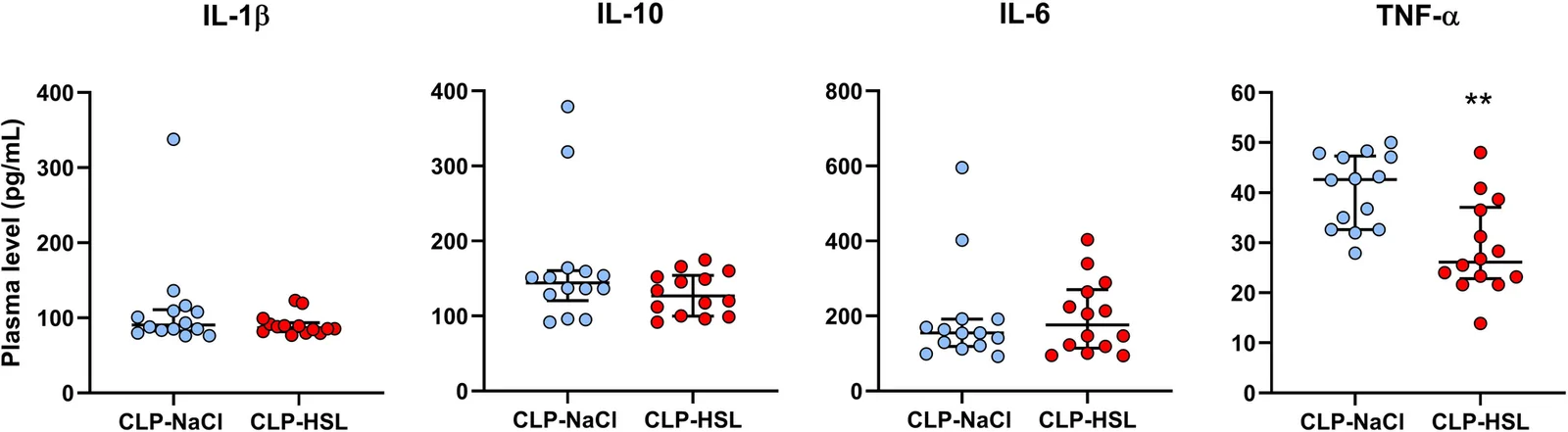
Blood inflammation. Plasma levels of cytokines Interleukine 1-β (IL-1β), Interleukine 10 (IL-10), Interleukine 6 (IL-6) and Tumor necrosis factor-alpha (TNF-α). CLP: Caecal ligation and puncture. HSL: Hypertonic sodium lactate. NaCl: Sodium chloride. *n* = 14 per group, **: *p* = 0.001, Mann-Whitney test

No significant differences were observed in plasma levels of IL-1β, IL-10 and IL-6 between the CLP-NaCl and CLP-HSL groups (Fig. 1).

In contrast, a reduction in TNF-α plasma level of around 40% was observed in the CLP-HSL group compared to CLP-NaCl (26.2 [22.8–37.1] vs. 42.6 [32.6–47.3] pg/mL, *p* = 0.001).

### Microcirculatory function

#### *In vivo* mesenteric microcirculation

**Fig. 2:**
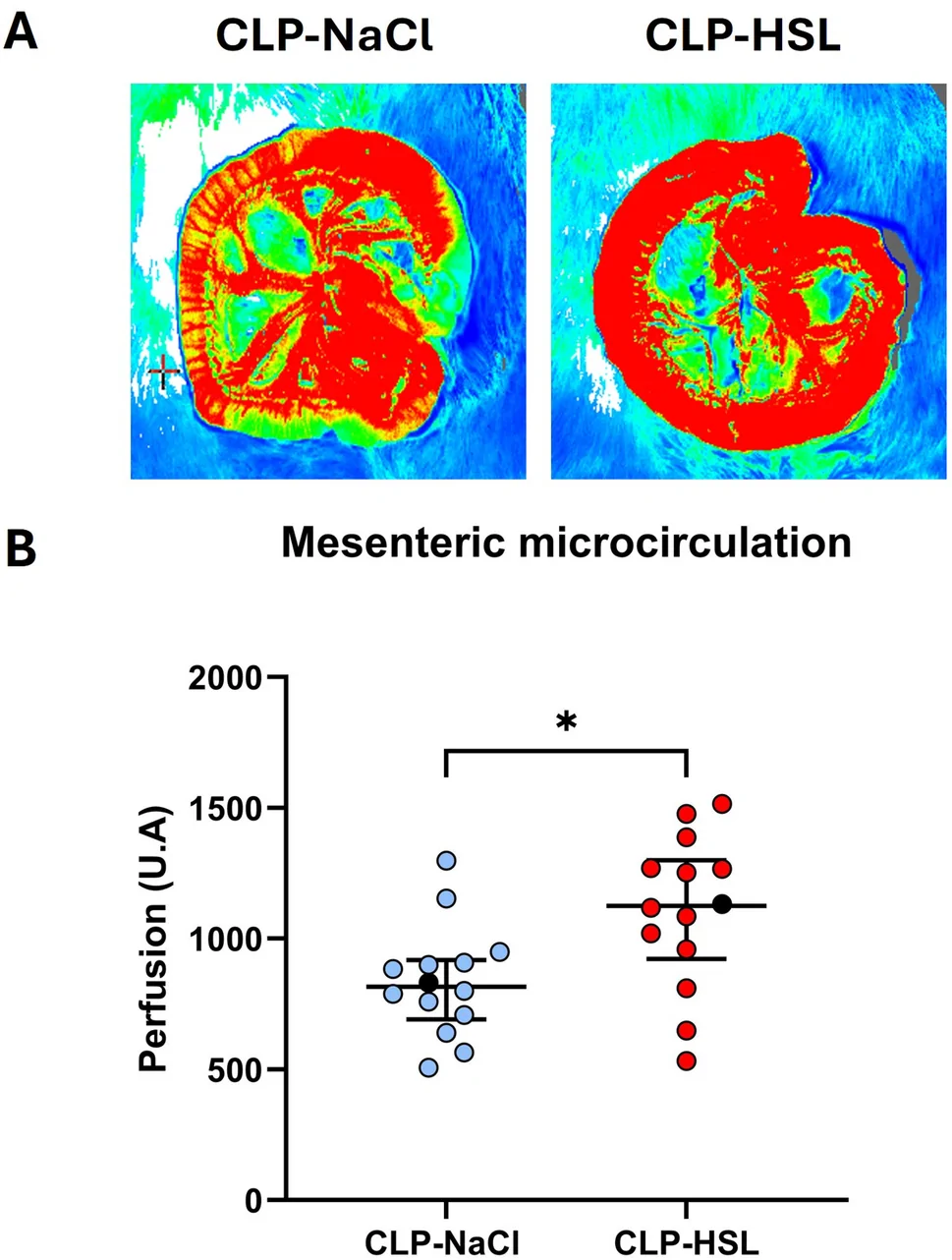
Mesenteric microcirculation. (**A**) Representative panel showing mesenteric microcirculatory blood flow assessed by laser speckle imaging from one animal per group corresponding to the black dot shown in (**B**). (**B**) Mesenteric perfusion in CLP-NaCl and CLP-HSL groups. CLP: Caecal ligation and puncture. HSL: Hypertonic sodium lactate. NaCl: Sodium chloride. *n* = 14 per group, *: *p* = 0.012, Mann-Whitney test

Mesenteric perfusion was significantly and greatly improved in the CLP-HSL group compared to the CLP-NaCl group (816.5 [692–919] vs. 1126 [923–1300] PU, *p* = 0.012) (Fig. 2).

To further investigate the role of lactate in microcirculatory function under inflammatory conditions, we examined its effects on ex vivo vascular reactivity using mesenteric arteriography.

#### Ex vivo mesenteric artery vasoreactivity assay

##### LPS impact on vasocontractility

**Fig. 3:**
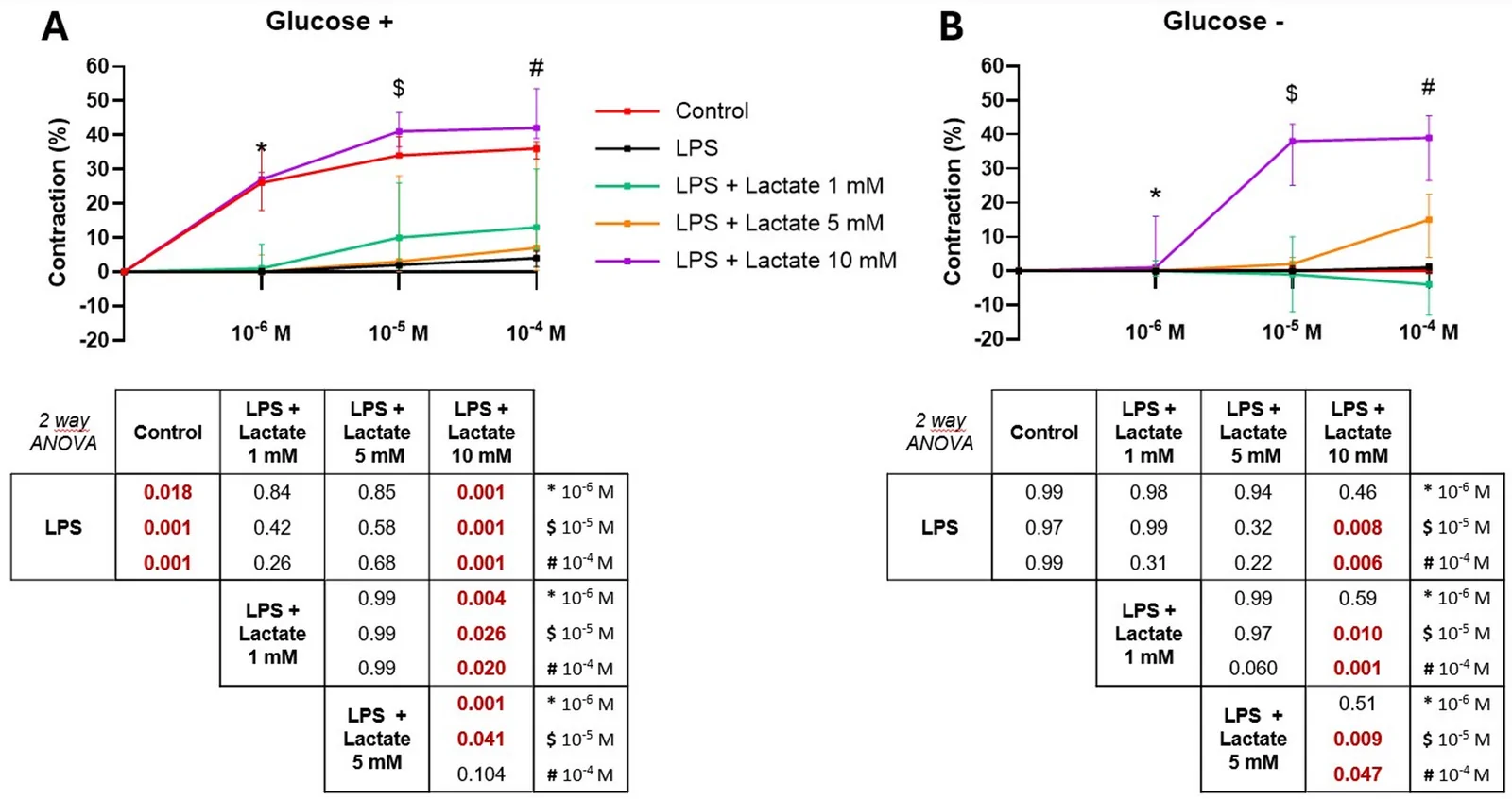
Effects of sodium lactate on ex vivo mesenteric arterial contractility under inflammatory conditions. Ex vivo assessment of mesenteric arterial contractility in response to increasing concentrations of phenylephrine (10⁻⁶ M, 10⁻⁵ M, and 10⁻⁴ M) under inflammatory condition (LPS) in the presence (**A**, Glucose +) or absence of glucose (**B**, Glucose –). *n* = 5 per group. LPS: Lipopolysaccharide. The accompanying tables summarize the *P* values for each statistical comparison performed using two-way ANOVA followed by Tukey’s post hoc multiple comparisons test

The mesenteric arteries were removed from non-sepsis rats and submitted to the exposure of LPS as an inflammatory stimulation. The results are presented in Fig. 3 and annex 3 A and 3B.

LPS significantly impaired arterial vasoconstriction in response to phenylephrine in the presence of glucose (normal conditions) compared to untreated controls. In contrast, when glucose was absent (deprivation conditions), the vasoconstrictive response to phenylephrine was nearly abolished in both control and LPS-treated groups, even at the highest phenylephrine concentrations tested, indicating that glucose availability is essential for maintaining vascular tone under these conditions.

##### Impact of sodium lactate on vasocontractility in inflammatory conditions

Under inflammatory conditions and in the presence of glucose, sodium lactate significantly improved vasoconstriction in a concentration-dependent manner. The 10 mM lactate treatment restored vascular tone across all phenylephrine concentrations (ranging from the lowest 10^− 6^M to the highest 10^− 4^M), with contractions similar to non-inflammatory controls. Conversely, reduced lactate concentrations of 1 and 5 mM were not associated with an improvement in contraction in comparison with LPS-controls.

##### Impact of sodium lactate on vasocontractility in absence of glucose and inflammatory conditions

Under combined conditions of glucose deprivation and LPS exposure, highest concentration of lactate (10 mM) was able to restore vasoconstriction at 10⁻⁵ M and 10⁻⁴ M phenylephrine, even compared with control artery unexposed to LPS.

## Discussion

We demonstrated that, although HSL exerts effects on macrocirculation comparable to those of NaCl, it improves microcirculatory perfusion, vascular function, and the inflammatory response in a resuscitated rat model of sepsis. These results reinforce previously published data and provide new insights by specifically evaluating microcirculation independently from macrocirculation, demonstrating a direct effect of HSL on resistance vessels. Moreover, we show that lactate can serve as an alternative to glucose under conditions of glucose deprivation, demonstrating its metabolic relevance when glucose availability is insufficient for cellular function—whether due to absolute deprivation (hypoglycemia) or insulin resistance, which is frequently observed during inflammation and sepsis. Finally, our *in vivo* model strengthens translational relevance by initiating fluid administration only when animals exhibited clinical signs of infection, whereas most prior studies initiated fluids either before sepsis induction (as in our previously published model) or at an arbitrary time point.

### Impact on macro and micro-circulation

HSL and NaCl exerted similar effects on macro-circulation, with restoration of blood pressure and similar heart function as explored by echocardiography and intracardiac pressure-volume analysis. In the context of sepsis resuscitation, where hemodynamic dysfunction is a central concern [3, 23, 26], HSL appears to be as efficient as NaCl in restoring systemic circulation. Previous studies have reported beneficial effects of HSL on circulation in more severe models of sepsis and endotoxemia [23, 24].

The absence of an additional circulation benefit compared with the CLP-NaCl group may be related to antibiotic treatment, which may have attenuated sepsis severity in the present model compared with our previous studies and thereby reduced fluid requirement. Importantly, the lack of difference in macro-circulation supports our question, as a difference is still observed in microcirculation* in vivo*.

Indeed, mesenteric microcirculation, a vascular territory highly sensitive to sepsis-induced perfusion deficits, was strongly improved. The occurrence of this effect independently of systemic hemodynamic changes is consistent with the concept of hemodynamic incoherence in sepsis [27–29], whereby micro- and macro-circulation can evolve in different ways.

We hypothesized that HSL presented specific beneficial microcirculatory effects, which have been explored in a second time by ex vivo arteriography.

### Vascular reactivity

To further investigate the microvascular effects of lactate, we used an ex vivo model of LPS-induced inflammation. These findings suggest that sodium lactate enhances vascular reactivity in a concentration-dependent manner under both control and inflammatory conditions. The ability of lactate to restore vasoconstriction following LPS-induced inflammation supports the concept that lactate can serve as an alternative metabolic substrate to sustain vascular function under inflammatory stress. Interestingly, the apparent difference between glucose-containing and glucose-free conditions may reflect differences in substrate availability. In the presence of glucose, vascular smooth muscle cells may preferentially rely on glucose metabolism, thereby reducing their dependence on exogenous lactate and attenuating the response observed at 5 mM. Conversely, under glucose-free conditions, lactate may be utilized to a greater extent as an alternative energy substrate, contributing to the more progressive concentration-dependent response. Furthermore, the absence of vasoconstriction in glucose-deprived conditions without lactate indicates that glucose is normally required to sustain vascular contraction, highlighting the ability of lactate to substitute for glucose and maintain vascular function under these metabolically compromised conditions.

### Effects on inflammation

Our results demonstrate a significant reduction in circulating TNF-α levels, whereas IL-6 concentrations remained unaffected. TNF-α is an early pro-inflammatory cytokine rapidly induced following pathogen recognition, whereas IL-6 is more associated with tissue-injuries, hypoperfusion and their damage-associated patterns [30–32]. In this context, the selective reduction in TNF-α plasma levels may suggest a modulation of pathogen-driven inflammatory signalling. However, further mechanistic studies are therefore needed to clarify the pathways involved. One potential mechanism underlying these effects may involve lactate-mediated receptor signalling and its anti-inflammatory proprieties.

The lactate concentrations used in our experiments fall within the EC₅₀ range for activation of GPR81 [33], a natural lactate receptor expressed in vascular and immune cells. GPR81 signalling has been implicated in anti-inflammatory responses and modulation of vascular tone [34–37]. By inhibiting the cAMP/PKA pathway, GPR81 activation may influence both vascular tone and endothelial barrier function, providing a mechanistic basis for lactate-mediated modulation of microcirculatory perfusion during sepsis. Moreover, modulation of GPR81 has been documented as modulating inflammatory responses both in sepsis and non-sepsis models, and this effect was highlighted in our study by reducing TNF-α plasma levels [24, 36, 38]. The hypothesis of GPR81 involvement warrants further mechanistic investigation, as it may represent a novel therapeutic axis linking metabolism and vascular function in sepsis. Thus, the restoration of vascular tone may have been, at least partially, explained by the reduction of inflammation. But beyond this effect, the fact that HSL was able to restore vascular tone in the absence of glucose, whereas non-sepsis control arteries were atoned, is also in favour for an independent and energetic mechanism.

### Metabolic effects

Although hypertonic sodium lactate infusion provides an exogenous lactate load, several of our findings suggest that the infused lactate was metabolized rather than accumulating in our model.

First, the reduction of plasma potassium, in association with a rise in TCO_2_, reflects the alkalinizing effect of lactate metabolism. According to physicochemical acid-base principles, metabolization of lactate increases the effective strong ion difference, leading to proton consumption and a shift toward alkalosis, frequently associated with intracellular potassium shift [39, 40]. The absence of effect on pH may be due by a compensatory change in frequency or tidal volume.

Second, the increase in acetoacetate (and maybe β-hydroxybutyrate, despite not reaching statistical significance), suggests enhanced oxidative metabolism of lactate. Lactate is transported into cells and converted into pyruvate by lactate dehydrogenase, which constitutes the first step toward mitochondrial oxidation [15, 41, 42]. Pyruvate can then generate acetyl-CoA, a substrate for the tricarboxylic acid cycle or for ketogenesis, leading to production of ketone bodies such as acetoacetate and β-hydroxybutyrate [43]. The produced ketone bodies (acetoacetate and β-hydroxybutyrate) are efficient oxidative fuels that may be integrated into the citric acid cycle in non-hepatic tissues. In support of the physiological relevance of this pathway, infusion of the ketone body 3-hydroxybutyrate in patients with chronic heart failure has been shown to increase cardiac output, highlighting the potential benefit of alternative metabolic substrate supplementation for circulatory support [44].

In addition, plasma glucose also tended to increase, although without reaching statistical significance. Lactate can be converted into glucose in the liver and kidneys via the Cori cycle, enabling gluconeogenesis and contributing to systemic energy availability and storage [45].

These findings support the concept that lactate can act as an essential energy substrate in sepsis, especially when glucose availability is limited or glucose utilization is impaired by insulin resistance [46].

### Strengths and limitations

This study has several notable strengths. It is the first to test HSL in a resuscitated CLP model, integrating fluid and antibiotic therapy and thereby closely mimicking clinical management. To ascertain the circulatory impact of our model, and therefore the septic status of the infection, we first characterized the circulatory profile using telemetry measurements. Our model is representative of a milder stage of sepsis, and continuous telemetry enabled us to identify the onset of clinical deterioration, considered to be the appropriate time point to initiate antibiotic therapy and fluid resuscitation, in accordance with current clinical practice. This reinforces the transposition to humans. The experimental approach was comprehensive, combining *in vivo* macro- and microcirculatory assessment, ex vivo vascular reactivity testing, and metabolic and inflammatory profiling. Furthermore, demonstrating lactate’s efficacy under glucose deprivation reinforces its relevance as an alternative energy source during septic metabolic stress [14, 23].

Limitations include the composition of the infused solutions. In particular, the HSL group received a higher sodium load and a more hypertonic solution compared with the NaCl group. These differences may influence fluid distribution, vascular tone, and hemodynamics, and therefore represent a potential confounding factor. However, despite the higher sodium content and osmolarity of HSL, we did not observe evidence of enhanced intravascular volume expansion. On the contrary, hematocrit levels were significantly higher in the HSL group, suggesting the absence of sustained plasma dilution induced by NaCl fluid. This finding argues against a predominant role of sodium-driven fluid shifts in explaining our results. Moreover, these findings may even be consistent with a chloride-driven reduction in plasma volume, as plasma chloride concentrations were lower in the HSL-treated group. This hypothesis is also supported by other studies showing that HSL induced negative chloride balance [23, 47, 48].

Taken together, these observations support the hypothesis that the effects of HSL are not solely attributable to its sodium content or osmotic properties but may involve specific metabolic and microvascular mechanisms related to lactate. Additionally, we did not include hypertonic NaHCO₃ control group, as this compound due to his known toxicity in this model [24].

Moreover, microcirculatory measurements were limited to the mesenteric bed, leaving other critical territories such as renal [49, 50] or coronary [51–53] circulation unassessed. Additionally, while metabolic data strongly suggest lactate utilization, confirmation with mitochondrial respiration measurements or isotope tracer studies could be performed.

Finally, in our *in vivo* experiments, measurements were performed in separate groups of animals because of experimental constraints and to preserve the quality and reliability of the data collected for each endpoint. Consequently, this experimental design may limit the direct integration of the different datasets.

In our vascular ex vivo experiment, the induction of inflammatory stress is achieved through the static incubation with LPS. This model presents the advantage of limiting the confusion bias related to systemic inflammation and to the inter-connection between macro- and micro-circulation, allowing us to specifically examine micro-vessels in a standardized environment. Nevertheless, the us LPS, which mainly stimulates the Tool-Like Receptor-4 way, may have limited the clinical relevance, as it does not reproduce the complexity of inflammatory mediators specific to sepsis, which are themselves major contributors to the alteration of vasomotricity [54].

## Conclusion

We demonstrated that HSL improves mesenteric microcirculation and reduces systemic inflammation and can serve as an alternative to glucose for vascular function a rat model of sepsis. These beneficial effects of HSL are secondary to intricated mechanisms, including direct metabolic effects, reduced inflammation and possibly osmotic effects. At the same time, HSL appears to be safe in our model. These results support the role of HSL as a promising candidate for sepsis resuscitation, warranting further evaluation in advanced preclinical models and clinical trials.

### Take-home message

Hypertonic sodium lactate dose-dependently improved mesenteric microcirculation and reduced inflammation, independently from macrocirculatory changes. Lactate may serve as a surrogate of glucose for small arteries.

## Supplementary Information

Below is the link to the electronic supplementary material.

Supplementary Material 1

Supplementary Material 2

Supplementary Material 3

## Data Availability

The datasets and materials are available from the corresponding author on reasonable request.

## Abbreviations

AWs: Anterior wall thickness at systole
AWd: Anterior wall thickness at diastole
CLP: Cecal ligation and puncture
CO: Cardiac output
FS: Fractional shortening
HSL: Hypertonic sodium lactate
IL-1β: Interleukin-1 beta
IL-6: Interleukin-6
IL-10: Interleukin-10
LPS: Lipopolysaccharide
LV: Left ventricle
LVED: Left ventricular end-diastolic diameter
LVEDP: Left ventricular end-diastolic pressure
LVEDPR: Left ventricular end-diastolic pressure–volume relation
LVESP: Left ventricular end-systolic pressure
LVESPVR: Left ventricular end-systolic pressure–volume relation
LVSF: Left ventricular shortening fraction
PaCO₂: Partial pressure of carbon dioxide in arterial blood
PaO₂: Partial pressure of oxygen in arterial blood
PU: Perfusion unit
PWd: Posterior wall thickness at diastole
PWs: Posterior wall thickness at systole
SV: Stroke volume
TCO₂: Total carbon dioxide content
TNF-α: Tumor necrosis factor-alpha
VTI: Velocity–time integral
