# Supplementary material for: Hypertonic sodium lactate improves microcirculation, modulates inflammation, and preserves vascular reactivity in a rat model of sepsis: in vivo and ex vivo evidence
Source: Intensive Care Med Exp. 2026 Sep 20;14:123. doi: 10.1186/s40635-026-00975-5 (PMC13590461; doi:10.1186/s40635-026-00975-5)
Supplement: Supplementary file 1 [file 40635_2026_975_MOESM1_ESM.docx]

**Annex 1. Experimental protocol.** CLP: Caecal ligation and puncture. D: day. H: Hours. HSL: Hypertonic sodium lactate. LV: Left ventricular. NaCl: Sodium chloride.
