# Supplementary material for: Hypertonic sodium lactate improves microcirculation, modulates inflammation, and preserves vascular reactivity in a rat model of sepsis: in vivo and ex vivo evidence
Source: Intensive Care Med Exp. 2026 Sep 20;14:123. doi: 10.1186/s40635-026-00975-5 (PMC13590461; doi:10.1186/s40635-026-00975-5)
Supplement: Supplementary file 2 [file 40635_2026_975_MOESM2_ESM.docx]

 **Annex 2A:** **Evolution of systolic and diastolic blood pressures, heart rate and survival** in the first 28 hours after induction of sepsis by caecal ligation and puncture (CLP) (black lines). Sham rats are represented in green lines. CLP: Caecal ligation and puncture. HSL: Hypertonic sodium lactate. NaCl: Sodium chloride. *: *p*=0.001 Sham vs CLP, Kruskal–Wallis’s test followed by Dunn's multiple comparisons test.

 **Annex 2B: Evolution of systolic and diastolic blood pressures, heart rate and survival** in the first 28 hours after induction of sepsis by caecal ligation and puncture (CLP) without (black lines) or with fluid infusion (NaCl infusion in blue lines, and HSL infusion in ref lines). Sham rats are represented in green lines. CLP: Caecal ligation and puncture. HSL: Hypertonic sodium lactate. NaCl: Sodium chloride. *: *p*=0.001 Sham *vs* CLP, $: *p*=0.01 CLP *vs* CLP-NaCl, #: *p*=0.01 CLP *vs* CLP-HSL, Kruskal–Wallis’s test followed by Dunn's multiple comparisons test.
