# Supplementary material for: Hypertonic sodium lactate improves microcirculation, modulates inflammation, and preserves vascular reactivity in a rat model of sepsis: in vivo and ex vivo evidence
Source: Intensive Care Med Exp. 2026 Sep 20;14:123. doi: 10.1186/s40635-026-00975-5 (PMC13590461; doi:10.1186/s40635-026-00975-5)
Supplement: Supplementary file 3 [file 40635_2026_975_MOESM3_ESM.docx]

|  | Phenylephrine **10^-6^M** | | Phenylephrine **10^-5^M** | | Phenylephrine **10^-4^M** | |
| --- | --- | --- | --- | --- | --- | --- |
|  | **Indiv. values** | Median  [Inter] | **Indiv. values** | Median  [Inter] | **Indiv. values** | Median  [Inter] |
| **Glucose -** |  |  |  |  |  |  |
| Control | **1** | 0.0  [-0.5-0.5] | **2** | 0.0  [-0.5-1.0] | **3** | 0.0  [-0.5-2.0] |
|  | **0** |  | **-1** |  | **-1** |  |
|  | **0** |  | **0** |  | **0** |  |
|  | **-1** |  | **0** |  | **0** |  |
|  | **0** |  | **0** |  | **-1** |  |
| LPS | **0** | 0.0  [-1.0-0.5] | **0** | 0.0  [0.0-1.5] | **1** | 1.0  [-0.5-2.0] |
|  | **-1** |  | **0** |  | **-1** |  |
|  | **-1** |  | **0** |  | **0** |  |
|  | **1** |  | **0** |  | **1** |  |
|  | **0** |  | **2** |  | **3** |  |
| LPS + Lactate  1 mM | **6** | 0.0  [-1.5-3.0] | **20** | -1.0  [-12.0-10.0] | **-4** | -4.0  [-13.0-0.0] |
|  | **0** |  | **-12** |  | **-12** |  |
|  | **-3** |  | **-12** |  | **-14** |  |
|  | **0** |  | **-1** |  | **0** |  |
|  | **0** |  | **0** |  | **0** |  |
| LPS + Lactate  5 mM | **-1** | 0.0  [-0.5-1.0] | **0** | 2.0  [1.0-4.0] | **5** | 15.0  [4.0-22.5] |
|  | **1** |  | **4** |  | **30** |  |
|  | **0** |  | **2** |  | **15** |  |
|  | **1** |  | **4** |  | **3** |  |
|  | **0** |  | **2** |  | **15** |  |
| LPS + Lactate  10 mM | **18** | 1.0  [0.5-16.0] | **47** | 38.0 [25.0-43.0] | **48** | 39.0  [26.5-45.5] |
|  | **14** |  | **38** |  | **39** |  |
|  | **0** |  | **30** |  | **23** |  |
|  | **1** |  | **20** |  | **30** |  |
|  | **1** |  | **39** |  | **43** |  |

**Annex 3A:** **Supplementary Table 1. Individual values (Indiv. values) and median with interquartile (median [inter]) of mesenteric arterial contraction (%)** in response to increasing concentrations of phenylephrine (10⁻⁶ M, 10⁻⁵ M, and 10⁻⁴ M) under inflammatory conditions (LPS), in the presence of glucose (Glucose +). n = 5 per group. Statistical analyses between groups were performed using a 2-way ANOVA for repeated measures including groups (control, LPS and LPS with increasing doses of Lactate) and Phenylephrine concentrations. Multiple comparisons were corrected using the Tukey’s test. LPS: Lipopolysaccharide.

|  | Phenylephrine **10^-6^M** | | Phenylephrine **10^-5^M** | | Phenylephrine **10^-4^M** | |
| --- | --- | --- | --- | --- | --- | --- |
|  | **Indiv. values** | Median  [Inter] | **Indiv. values** | Median  [Inter] | **Indiv. values** | Median  [Inter] |
| **Glucose +** |  |  |  |  |  |  |
| Control | **26** | 26.0  [18.0-36.0] | **34** | 34.0  [34.0-39.5] | **34** | 36.0  [33.0-38.0] |
|  | **36** |  | **34** |  | **32** |  |
|  | **36** |  | **41** |  | **39** |  |
|  | **24** |  | **38** |  | **36** |  |
|  | **12** |  | **34** |  | **37** |  |
| LPS | **0** | 0.0  [-0.5-1] | **2** | 2.0  [0.5-2.0] | **5** | 4.0  [1.5-6.0] |
|  | **0** |  | **2** |  | **7** |  |
|  | **1** |  | **0** |  | **4** |  |
|  | **1** |  | **1** |  | **4** |  |
|  | **-1** |  | **2** |  | **-1** |  |
| LPS + Lactate  1 mM | **15** | 1.0  [-0.5-8.0] | **22** | 10.0  [1.0-26.0] | **8** | 13.0  [6.5-30.0] |
|  | **1** |  | **10** |  | **13** |  |
|  | **-1** |  | **-1** |  | **31** |  |
|  | **0** |  | **3** |  | **5** |  |
|  | **1** |  | **30** |  | **29** |  |
| LPS + Lactate  5 mM | **0** | 0.0  [0.0-5.0] | **0** | 3.0  [0.0-28.0] | **0** | 7.0  [0.5-35.0] |
|  | **1** |  | **0** |  | **1** |  |
|  | **0** |  | **3** |  | **7** |  |
|  | **0** |  | **29** |  | **45** |  |
|  | **9** |  | **27** |  | **25** |  |
| LPS + Lactate  10 mM | **27** | 27.0  [26.0-29.0] | **41** | 41.0  [36.5-46.5] | **42** | 42.0  [39.0-53.5] |
|  | **27** |  | **36** |  | **54** |  |
|  | **27** |  | **52** |  | **53** |  |
|  | **31** |  | **41** |  | **41** |  |
|  | **25** |  | **37** |  | **37** |  |

**Annex 3B:** **Supplementary Table 2. Individual values (Indiv. values) and median with interquartile (median [inter]) of mesenteric arterial contraction (%)** in response to increasing concentrations of phenylephrine (10⁻⁶ M, 10⁻⁵ M, and 10⁻⁴ M) under inflammatory conditions (LPS), in the absence of glucose (Glucose −). n = 5 per group. Statistical analyses between groups were performed using a 2-way ANOVA for repeated measures including groups (control, LPS and LPS with increasing doses of Lactate) and Phenylephrine concentrations. Multiple comparisons were corrected using the Tukey’s test. LPS: Lipopolysaccharide.
